# Baseline Gene Expression Levels in Falkland-Malvinas Island Penguins: Towards a New Monitoring Paradigm

**DOI:** 10.3390/life12020258

**Published:** 2022-02-09

**Authors:** Lizabeth Bowen, Shannon Waters, Jeffrey L. Stott, Ann Duncan, Randi Meyerson, Sarah Woodhouse

**Affiliations:** 1 U.S. Geological Survey, Western Ecological Research Center, One Shields Avenue, Davis, CA 95616, USA; swaters@usgs.gov; 2 Department of Pathology, Microbiology and Immunology, University of California, One Shields Avenue, Davis, CA 95616, USA; jlstott@ucdavis.edu; 3 Detroit Zoo, 8450 W. 10 Mile Road, Royal Oak, MI 48067, USA; aduncan@dzs.org; 4 Independent Researcher, Farmington, MI 48332, USA; randimeyerson61@gmail.com; 5 Henry Doorly Zoo and Aquarium, 3701 S 10th St, Omaha, NE 68107, USA; Sarah.Woodhouse@omahazoo.com

**Keywords:** transcriptomics, penguins, wildlife monitoring, Falkland-Malvinas Islands

## Abstract

Health diagnostics of wildlife have historically relied on the evaluation of select serum biomarkers and the identification of a contaminant or pathogen burden within specific tissues as an indicator of a level of insult. However, these approaches fail to measure the physiological reaction of the individual to stressors, thus limiting the scope of interpretation. Gene-based health diagnostics provide an opportunity for an alternate, whole-system, or holistic assessment of health, not only in individuals or populations but potentially in ecosystems. Seabirds are among the most threatened marine taxonomic groups in the world, with ~25% of this species currently listed as threatened or considered of special concern; among seabirds, the penguins (Family Spheniscidae) are the most threatened seabird Family. We used gene expression to develop baseline physiological indices for wild penguins in the Falkland-Malvinas Islands, and captive zoo penguins. We identified the almost complete statistical separation of penguin groups (gentoo Detroit Zoo, gentoo Falkland-Malvinas Islands, rockhopper Detroit Zoo, and rockhopper Falkland-Malvinas Islands) based on gene expression profiles. Implementation of long-term longitudinal studies would allow for the assessment of temporal increases or decreases of select transcripts and would facilitate interpretation of the drivers of change.

## 1. Introduction

Traditional evaluation of the health status of wildlife is based on a combination of the animal’s history (e.g., movement, reproductive status), physical examination, and clinical pathology data. Many studies focusing on sensitive populations are disease-centered, while relatively few studies focus on differences in host susceptibility, which may be due to differences in the host immune response [1] and can be influenced by factors such as nutrition, contaminants, and pathogens [2]. Additionally, although the exact cause of most species declines is unknown, declines are likely associated with multiple and potentially synergistic environmental stressors. Historically, large-scale investigations into populations and ecosystems have been driven by species declines and/or mortality events. However, by the time these events are observed, ample time has elapsed in systems already operating sub-optimally for additional conditions to manifest, preventing clear insight into the causal factors. Alternatively, using a proactive approach of baseline and long-term monitoring to continually assess populations for subtle yet significant changes would provide baseline data sets upon which perturbations in real time could be assessed.

Gene-based techniques have the ability to improve our understanding of the stressor/immune function/disease triad by measuring the physiological response of individuals to disease as well as the influence of external or environmental factors on the health of an individual and, collectively, the population. Gene expression is the process by which information from the DNA template of a particular gene is transcribed into messenger RNA (mRNA) and eventually translated into a functional protein. The earliest observable signs of health impairment are altered levels of gene transcripts that are evident prior to clinical manifestation [3]. The amount of a particular gene that is transcribed is physiologically dictated by a number of intrinsic and extrinsic factors, including stimuli such as infectious agents, contaminant exposure, nutritional deficit, or trauma [4,5,6,7]. The use of novel molecular techniques may help identify these and other factors contributing to host susceptibility, which is critical to developing strategies to mitigate the effect of stressors on wildlife populations [8].

As an example, seabirds have a global distribution, are vital components of coastal marine ecosystems, and may connect marine and terrestrial environments at a global scale [9,10,11]. Because seabirds are dependent on a variety of ecosystems, they are vulnerable to both marine and terrestrial environmental stressors and the synergistic effects of combined stressors [11]. Not surprisingly, seabirds are the most threatened marine taxonomic group in the world, with ~25% of this species currently listed as threatened or considered of special concern [12]. Among seabirds, the penguins (Family Spheniscidae) are the most threatened seabird Family [12]. Populations of many penguin species have declined substantially in the past two decades. In 2013, 11 species (60%) were listed as threatened (five endangered and six vulnerable), two as near-threatened, and five as of least concern [13]. The Falkland-Malvinas Islands are a marine biodiversity hotspot and important for penguin conservation because they have important breeding populations of four penguin species (king (*Aptenodytes patagonicus*), gentoo (*Pygoscelis papua*), southern rockhopper (*Eudyptes chrysocome*), and Magellanic (*Spheniscus magellanicus*)), with the largest breeding populations of southern rockhopper and gentoo penguins [14]. The abundance of penguins in the Falkland-Malvinas Islands declined by 84% during the 1980s and 1990s. Southern rockhopper penguins are the most threatened penguin in the Falkland-Malvinas Islands (vulnerable on the IUCN Redlist; [15]) and are considered threatened under the US Endangered Species Act. Gentoos are considered near-threatened, with a decreasing population (IUCN Redlist; [15]).

The broad goal of our project was to develop the methodology for and provide a baseline assessment of select gene expression levels in penguins in the Falkland-Malvinas Islands. This baseline will be a reference from which future measurements, and thus subtle yet significant alterations in physiological status, can be made in real time, prior to catastrophic events. We compared the expression of targeted genes within and between gentoo and rockhopper penguin populations sampled in the Falkland-Malvinas Islands and between these penguins and captive penguins of both species. With these assays, we provide initial transcript-based analyses and results for penguin populations that can be used as reference to help identify individual, population, and ecosystem changes in the future [5]. These assays can be adapted globally and across species, and ultimately may provide early-warning indicators and help us better understand the susceptibility of individuals, populations, and ecosystems to risks from changing conditions. Similar gene expression profiling has been adapted for use with multiple and divergent species and has also been used successfully as a predictor of mortality [4,5,6,7].

## 2. Materials and Methods

### 2.1. Falkland-Malvinas Islands

**Figure 1:**
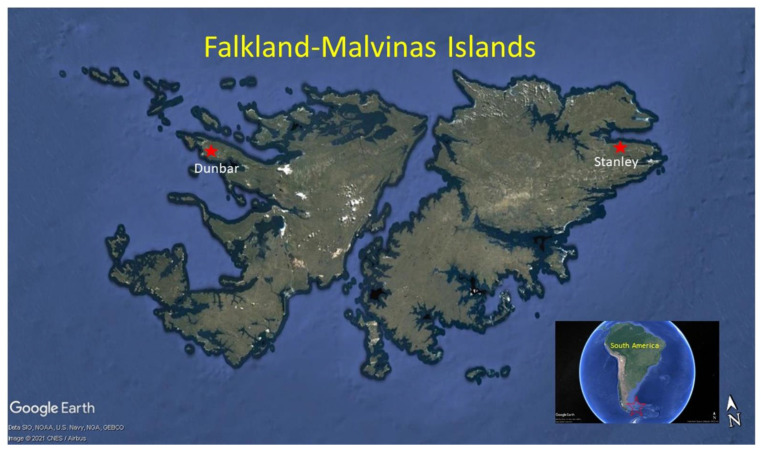
Gentoo and rockhopper penguin sampling sites at two colonies in the Falkland-Malvinas Islands (Dunbar and Stanley).

Fieldwork was conducted during the 2019 nesting season (late November to early December). We conducted health and welfare assessments for gentoo and rockhopper penguins at two study sites: Dunbar in the west Falkland-Malvinas (hereafter, islands), which has little shipping/oil development (~51°22′ S, 60°38′ W), and Berkeley Sound in the east islands near Stanley, which has heavy shipping activity (~S 51°33′, 57°46′ W, Figure 1). The two study sites are separated by distance and by the prevailing ocean currents, which flow in opposite directions at each site (Figure 1).

All handling was performed near the nesting sites, at a distance that did not elicit a response from the nesting penguins. We sampled 39 gentoo and 34 rockhopper penguins. Penguins were manually restrained by animal care staff from the Detroit Zoological Society who are experienced in handling penguins. All penguins in the study received a complete physical exam by a veterinarian. The time of manual restraint to complete the exam and sampling was 5–10 min per bird. Any abnormal findings were recorded. Samples were stored in a liquid nitrogen storage tank for 4–7 days before being transferred to a −80 °C freezer.

### 2.2. Detroit Zoo

We sampled 23 gentoo and 15 captive rockhopper penguins located at the Detroit Zoo, Michigan. Sampling did not occur during breeding season. All penguins of each species received the same diet/water quality/husbandry and lived in the same habitat. Penguins were manually restrained by animal care staff from the Detroit Zoological Society who are experienced in handling penguins. The time of manual restraint to complete measurements and sampling was 5–10 min per bird. All penguins in the study received a complete physical exam by a veterinarian. Any abnormal findings were recorded, and all birds were considered to be healthy. Each bird had been previously banded on the wings using color-coded bands.

### 2.3. Blood Collection and RNA Extraction

Blood was collected from the jugular vein using a 20-gauge needle. A 0.5-mL blood sample from each penguin was then placed into a blood collection tube with RNA preservative buffer (Captive: RNeasy Animal Protect, Qiagen, Valencia, CA, USA; Wild: DNA/RNA Shield Blood Collection Tubes, Zymo Research, Irvine, CA, USA), shipped at room temperature per manufacturer’s instructions, and stored at −20 °C prior to extraction of RNA [16]. RNA isolation methods do not influence quality or quantity of RNA [17]. We selected samples randomly for processing (i.e., samples were not processed in batches according to location, age, sex, or pathogen-exposure status).

### 2.4. Captive Penguins

Blood samples were placed directly into RNeasy Protect Animal Blood Tubes (Qiagen, Valencia, CA, USA) and then frozen at −20 °C until extraction of RNA [16]. Rapid RNA degradation and induced expression of certain genes after blood draws have led to the development of methodologies for preserving the RNA expression profile immediately after blood is drawn. The RNeasy Protect Animal Blood Tube contains a blend of RNA stabilizing reagents that protect RNA molecules from degradation by RNases and prevent further induction of gene expression. The RNA from blood in RNeasy Protect Animal Blood Tubes was isolated according to manufacturer’s standard protocols, except that each sample was initially split in half to account for the nucleated red blood cells. The extracted RNA was stored at −80 °C until analysis [18].

The RNA was then cleaned up using the Zymo RNA Clean and Concentrator-25 kit. The RNA from the previous procedure (60 µL) was added to 40 µL of molecular water to make a total of 100 µL, and then 200 µL of RNA binding buffer was added. The manufacture’s standard protocol (page 3) was followed, which included an in-column DNase treatment to remove contaminating gDNA. Extracted RNA was stored at −80 °C until analysis. We measured RNA concentration and clarity using a Qubit 3.0 Fluorometer with the Qubit Broad Range and the RNA, DNA, and RNA IQ Assay Kits (Life Technologies, Carlsbad, CA, USA).

### 2.5. Wild Penguins

The Zymo DNA/RNA Shield blood collection tubes were inverted 10 times prior to sample removal. RNA was extracted following modified procedures of Zymo Quick-RNA Whole Blood kit recommended by Zymo Technical Support. Following the Nucleated Whole Blood procedure on page 6 starting at step 2, the 100 µL sample was treated with 800 µL PK Digestion Buffer and 20 µL Proteinase K and then incubated at 55 °C for 30 min. Following this step, the sample was purified using the procedure on page 3 starting at step 3. The samples were treated with in-column DNase treatment to remove contaminating gDNA. Extracted RNA was stored at −80 °C until analysis. RNA concentration and clarity were measured on a Qubit 3.0 Fluorometer using the Qubit Broad Range with the RNA, DNA and RNA IQ Assay Kits (Life Technologies, Carlsbad, CA, USA).

### 2.6. cDNA Synthesis

We performed a standard cDNA synthesis on 2 ug of RNA template from each penguin. Reaction conditions included four units reverse transcriptase (Omniscript^®^; Qiagen, Valencia, CA, USA), 1 uM random hexamers, 0.5 mM each dNTP, and 10 units RNase inhibitor, in reverse transcription buffer (Qiagen). We incubated reactions for 60 min at 37 °C, followed by an enzyme inactivation step of 5 min at 93 °C, and then stored samples at −20 °C until further analysis.

### 2.7. Gene Selection

The genes examined in our study can be grouped into functional categories that include immune modulation, pathogen response, inflammation, cell signaling, xenobiotic-metabolizing enzymes, and cellular stress response, and were largely selected based upon their potential to be modified by biological, physical, or anthropogenic injury, thus providing information on the type and magnitude of stressors associated with the penguin’s internal or external environment (Table 1). Specifically, genes were chosen to reflect potential influences of known stressors in the penguins’ environments, both wild and captive. These include stressors associated with climate change (e.g., emerging infectious diseases, thermal stress, and nutritional stress), increased oil exploration (e.g., hydrocarbon exposure), and increasing tourism (e.g., general stress due to increased human presence). The functionality and response of each gene have been validated in other studies and are well-documented in the literature (Table 1).

### 2.8. Polymerase Chain Reaction Primers

We designed degenerate primers from multispecies alignments (GenBank) as previously described [46]. Briefly, we utilized degenerate primer pairs developed for the penguin on cDNA from 3 randomly selected gentoo and rockhopper samples. We designed degenerate primer pairs to amplify the genes of interest and two reference genes (Table 1; [46]). We performed PCR amplifications using these primers on 1 µL of each cDNA sample in 24.5 µL volume containing 20 µL of water, 2.5 µL 10× Advantage 2 PCR buffer, 0.5 µL 50× dNTP Mix, 0.5 µL of each primer, and 0.5 µL 50× Advantage 2 polymerase mix of Advantage^®^ 2 Taq polymerase (Clontech, Palo Alto, CA, USA). We performed the PCR on an BioRad C1000 Thermal Cycler (Hercules, CA, USA); it consisted of 1 cycle at 95 °C for 1 min, and then 40 cycles at 95 °C for 30 s, at 68 °C for 1 min, and 68 °C for 1 min; the ramp-up throughout was 1 °C/s, followed by a hold phase for 12 °C indefinitely. We electrophoresed the products of these reactions on 1.5% agarose gels and visualized resulting bands using BioRad U-View. We excised from the gel definitive bands representing PCR products of a predicted base-pair size of the targeted gene and extracted and purified them using a commercially available nucleic acid-binding resin (Zymoclean Gel DNA Recovery Kit). We determined nucleotide sequences of isolated fragments by dideoxy nucleotide methodology using an automated sequencer (Model 373; Applied Biosystems, Foster City, CA, USA). We analyzed nucleotide sequences of the PCR products using Align™ and Contig™ sequence-alignment software programs (Vector NTI™; Informax Inc., North Bethesda, MD, USA) and compared them with known sequences using the National Center for Biotechnology Information Basic Local Alignment Search Tool program [47], and with the IMGT/HLA database [48]. We designed primer pairs appropriate for real-time PCR based on the elucidated penguin sequences for each gene; we confirmed putative real-time primers on real-time PCR, purified the PCR product (Zymo DNA Clean and Concentrator-5 Kit), and sent the PCR product to sequencing for validation (Table 2).

### 2.9. Real-Time Polymerase Chain Reaction

We ran real-time PCR systems for the individual, gentoo, and rockhopper-specific reference genes and genes of interest in separate wells (Table 1). Briefly, we added 1 µL of cDNA to a mix containing 12.5 µL of QuantiTect Fast SYBR Green^®^ Master Mix (5 mM Mg^2+^) (Qiagen, Hilden, Germany), 0.5 µL each of forward and reverse sequence-specific primers, and 10.5 μL of RNase-free water; total reaction mixture was 25 µL. We loaded the reaction-mixture cDNA samples for each gene of interest and the reference genes into MicroAmp Fast Optical^®^ 96-well reaction plates in duplicate and sealed them with optical sealing tape (Applied Biosystems, Bedford, MA, USA). We used reaction mixtures containing water but no cDNA as negative controls; thus, we ran approximately two individual penguin samples per plate. We conducted amplifications on a QuantStudio 3 Real-time Thermal Cycler (Applied Biosystems, Bedford, MA, USA) using QuantStudio 3 Software. Reaction conditions were as follows: an initial hold stage of 95 °C for 20 s, 40 cycles of 95 °C for 1 s, and 60 °C for 20 s. The melt curve consisted of 95 °C for 1 s, 60 °C for 20 s, 0.3 °C/s temperature increase, and then 95 °C for 1 s. We evaluated the stability of reference genes (EF1a and YWHAZ) and ranked them using the web-based analysis tool RefFinder (https://www.heartcure.com.au/reffinder/ (accessed on 2 October 2020; [49]). We normalized cycle threshold crossing values (CT) for the genes of interest to the more stable of the reference genes, YWHAZ.

### 2.10. Statistical Methods

Our general approach was to evaluate the associations among gene expression levels, species, and locations. We analyzed qPCR data using normalized C_T_ values (housekeeping gene threshold crossing subtracted from the gene of interest threshold crossing); the lower the normalized value, the more transcripts are present. A change in normalized value of 2 is approximately equivalent to a 4-fold change in the amount of the transcript.

## 3. Results

### 3.1. General Description

**Figure 2:**
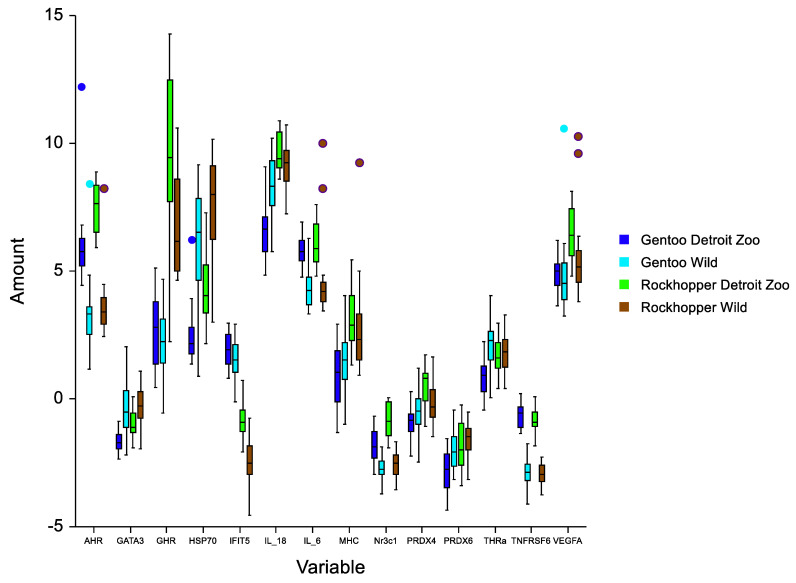
Distribution of average cycle threshold (CT) values across genes targeted by the panel of 14 primer pairs. Real-time PCR data are represented as normalized values (NVs); the lower the NV, the larger the quantity of transcripts. Blood was sampled from four groups of penguins during 2019 (gentoo Detroit Zoo, *n* = 23; gentoo Falkland-Malvinas Islands (Wild), *n* = 39; rockhopper Detroit Zoo, *n* = 15; rockhopper Falkland-Malvinas Islands (Wild), *n* = 34). Boxes are delineated by 25th and 75th percentiles. The 50th percentile median is indicated. Whisker length uses the classic method of box edge + (1.5; interquartile range), and severe outliers (circles) are calculated as box edge + (3; IQR). Interpretation of gene abbreviations is provided in Table 1.

We used a box-and-whisker plot to visually describe gene expression profiles by location (Figure 2). Although most population responses were overlapping to some degree, clear differences exist among responses for each location. Most genes showed fairly small ranges in expression; however, for growth hormone receptor (GHR), the range was relatively large, indicating a wider range of stimuli and responses (Figure 2). We calculated means and standard deviations for all variables across all sites and species (Table 3; Table S1). We used mixed-effects statistical models to simultaneously estimate and account for multiple influences (sex, location, and species) on gene expression levels, which make them appropriate for the multiple uncontrolled factors that occurred as a result of opportunistic samples acquired for this study (Table 4). We calculated parameter estimates for all model effects using the lme4 package in R 2.8.1 [50]. We calculated expression differences between species within the islands and Detroit Zoo as well as between rookeries at the islands using MANOVA (NCSS^©^ Statistical Software 2007, Kaysville, UT, USA). Significance was determined at *p* ≤ 0.05. Location and species had significant effects on the expression levels of most genes, while sex significantly influenced expression levels of 50% of the genes examined (Table 4).

**Figure 3:**
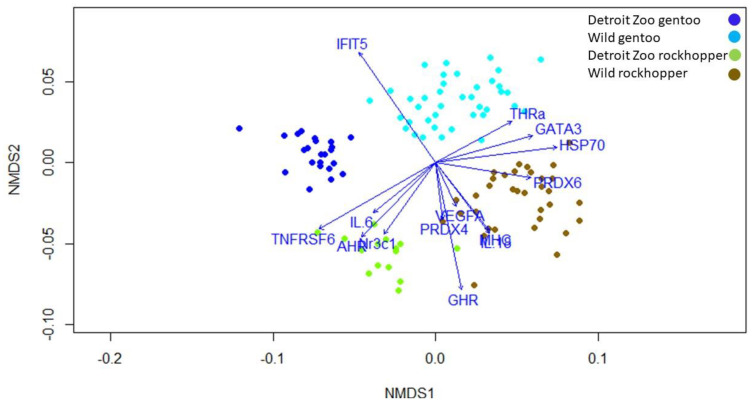
Two-dimensional non-parametric multidimensional scaling plot of the Bray–Curtis dissimilarity from gene transcript levels. The vector arrows signify the direction of maximum correlation for each gene in the ordination space and are significant at *p* < 0.05. The length of the arrows signifies the strength of the relationship of each metric and the two NMDS metrics, with longer arrows signifying greater strength. There is clear clustering by site. Results show that the separation of Detroit Zoo and Falkland-Malvinas (Wild) penguins is driven by higher levels of THRa, GATA3, PRDX6, and HSP70 in Detroit Zoo penguins and higher levels of TNFRSF6, AHR, IL-6, and Nr3c1 in Falkland-Malvinas Island penguins. The separation of rockhopper and gentoo penguins is driven by higher levels of IFIT5 in rockhopper penguins and higher levels of GHR, IL-18, VEGFA, PRDX4, and MHC in gentoo penguins.

We conducted two-dimensional non-parametric multidimensional scaling of the Bray-Curtis dissimilarity from gene transcripts using the Vegan package in R version 3.5.0. We obtained vectors describing the strength of each gene contribution to the two non-metric multidimensional scaling (NMDS) axes for graphical display. We evaluated goodness of fit for NMDS models using stress plots. The graphical representations show individual penguins clustered by similarity in expression values and not by pre-defined groups such as location. Penguins are separated into four well-defined groups (NMDS; 2D R^2^ = 0.98; Figure 3). Gene expression (C_T_) values differed among gentoo and rockhopper penguins sampled in the islands and at the Detroit Zoo (ANOSIM, *p* < 0.001, global R = 0.83) and were confirmed by cluster analysis (SIMPROF, *p* < 0.001). Vector analysis results show that the separation of Detroit Zoo and wild penguins is driven by higher levels of thyroid hormone receptor alpha (THRa), GATA3, peroxiredoxin 6 (PRDX6), and heat-shock protein 70 (HSP70) in Detroit Zoo penguins and higher levels of interleukin 6 (IL-6), tumor necrosis factor receptor super family 6 (TNFRSF6), aryl hydrocarbon receptor (AHR), and nuclear receptor subfamily 3 group C member 1 (Nr3c1) in wild penguins. The separation of rockhopper and gentoo penguins is driven by higher levels of interferon-induced protein with tetratricopeptide repeats 5 (IFIT5) in rockhopper penguins and higher levels of vascular endothelial growth factor A (VEGFA), peroxiredoxin 4 (PRDX4), GHR, IL-18, and major histocompatibility complex class II beta MHC in gentoo penguins.

### 3.2. Falkland-Malvinas Islands

We found gene expression differences between species in the islands. Expression was higher in gentoo penguins for GHR, HSP70, IL-18, MHC, and PRDX6, and higher in rockhopper penguins for IFIT5 (Table 3). We also found gene expression differences between colonies within gentoo penguins. Gene expression levels for IFIT5, IL-18, and PRDX4 were all higher at Stanley in comparison with Dunbar colonies. We found minimal gene expression differences between colonies within rockhopper penguins. HSP70 expression was higher in rockhopper penguins sampled at Dunbar, while TNFRSF6 expression was higher in rockhopper penguins sampled at Stanley (data not shown).

### 3.3. Detroit Zoo

We found gene expression differences between species at the Detroit Zoo. Expression was higher in gentoo penguins for AHR, GATA3, GHR, HSP70, IL-18, MHC, Nr3c1, PRDX4, PRDX6, THRa, and VEGFA, and higher in rockhopper penguins for IFIT5 (Table 3).

## 4. Discussion

Although the shortcomings of these analyses are evidenced by the small sample sizes and brief timeframe in which the animals were sampled, the methodology we developed and applied to penguins in the Falkland-Malvinas Islands is a baseline to which future measurements can be compared, and thus subtle yet significant alterations in physiological status can be identified in real time, prior to catastrophic events. On a broad scale, these methods can serve as a template across species and landscapes, potentially serving as early-warning indicators ecosystem-level disruptions. As well, the stark differences in gene expression patterns between species and locations is intriguing and warrants further study. Implementation of long-term longitudinal sampling and transcriptomics would allow assessment of temporal increases or decreases of select expression and would facilitate interpretation of the drivers of change and health within populations.

In this study, we identified near complete statistical separation of penguin groups (gentoo Detroit Zoo, gentoo islands, rockhopper Detroit Zoo, and rockhopper islands) based on gene expression profiles. Individuals within a wild population comprise a range of physiological states. Variation in gene expression occurs in healthy individuals and can be attributed to both intrinsic and extrinsic factors [3]. However, without reference ranges for the genes in our panel specific to the species analyzed, comparisons among groups are relative. Although significantly different expression values may still fall within the range of “normal”, differences are still indicative of differential intrinsic or extrinsic stimuli. One important difference between the sampled populations is age. Some captive penguins live into their 30s, and we can safely assume that the wild penguins are much younger. Additionally, penguins were sampled during breeding season in the Falkland-Malvinas Islands, while captive penguins were not sampled during breeding season. Although it is a logical conclusion that gene expression in some physiological systems may be altered by breeding status and/or age, the genes we have selected have not been examined in that light. While none of the genes in our panel is specific to ageing or reproduction, the complexities and interconnectedness of physiological systems in general require that we approach our results and interpretations with a degree of caution. Therefore, while we found expression differences between captive and wild penguins, we cannot, with certainty, attribute these differences to captive or wild environments; age and/or breeding status may contribute to expression differences.

### 4.1. Gene Expression Differences by Sex

Overall, females exhibited higher expression of AHR, IFIT5, interleukin 6 (IL-6), Nr3c1, TNFRSF6, and vascular endothelial growth factor A (VEGFA), while males exhibited higher expression of GHR. However, when analyzed separately by species and location, the effects of sex on gene expression were much lower. For example, sex had no influence on expression level among all genes in rockhopper penguins housed in the Detroit Zoo. Sex did influence expression levels of GHR and IL-18 (both higher in males) in gentoo penguins housed in the Detroit Zoo. As GHR expression was higher in male gentoo penguins in both locations, we would assume that GHR is reflective of a species-specific physiological adaptation. Females of both species sampled in the wild exhibited higher levels of genes indicative of stress (HSP70, rockhopper; Nr3c1, gentoo). In captive gentoo penguins, males exhibited higher levels of IL-18, which is involved in inflammatory responses to tissue injury and/or microbial presence.

### 4.2. Gene Expression Differences by Location

We found expression differences between wild penguins and those at the Detroit Zoo, regardless of species. Expression of the AHR gene was higher in wild penguins than those at the Detroit Zoo. The AHR responds to classes of environmental toxicants, including polycyclic aromatic hydrocarbons, polyhalogenated hydrocarbons, dibenzofurans, and dioxin [28]. The islands are becoming increasingly important for oil exploration, and there may already be abundant hydrocarbon presence in the environment from anthropogenic sources (including in prey items) to stimulate a detoxification response in penguins. At the same time, wild penguins exhibited higher levels of response to virus (IFIT5, TNFRSF6), response to tissue damage (including inflammation and stress; IL-6), and stress associated with cortisol production (Nr3c1). The combined effects of exposure to contaminants and pathogens may act synergistically to increase impacts to vulnerable populations. Penguins located at the Detroit Zoo, in comparison, expressed higher levels of HSP70. Additionally, penguins at the Detroit Zoo have evidence of higher responses to oxidative stress (PRDX6) and higher inflammatory responses (IL-18).

Within wild gentoo (IFIT5, IL-18) and rockhopper (TNFRSF6) penguins we found increased response to virus at Stanley in comparison with Dunbar. Gentoo penguins also exhibited increased oxidative stress (PRDX4) at Stanley. Rockhopper penguins exhibited and increased general stress (HSP70) response at Dunbar. In addition to the differences in shipping/oil development between Dunbar (little) and Stanley (heavy), most of the island’s human population live in Stanley. There are also approximately 2000 temporary inhabitants living on the Mount Pleasant military base to the south-west of Stanley. Without further investigation into rockhopper behavior patterns at Dunbar, it is difficult to explain the increased levels of stress response there.

### 4.3. Gene Expression Differences by Species

Although taxonomically similar at the family level, gentoo and southern rockhopper penguins maintain differences in biology and ecology and provide an interesting comparison on the potential effects of stressors and different routes for disease transmission. Gentoos are local foragers, while rockhoppers forage both locally and more distantly, which brings them into more potential contact with a variety of potential stressors, including hydrocarbons [14,51]. Gentoo and southern rockhopper penguins are genetically similar for the genes of interest in our study. Additionally, these two species inhabit the same habitat in the islands. Thus, the dramatic differences in gene expression between these two species in the wild would seem to be driven by differences in foraging strategies, prey items, and underlying physiological differences. While expression patterns in island gentoo penguins are indicative of relatively higher levels of nutrition, they also indicate increased levels of general stress and oxidative stress, accompanied by inflammation. Gentoo penguins also exhibit a higher response to bacterial pathogens. Rockhopper penguins in the islands exhibit a strong anti-viral response in comparison.

## 5. Conclusions

Population assessment and recovery planning focus on the inventory or number of animals; less attention is paid to identification of habitats, the effects of habitat fragmentation, and a more complete understanding of the quality or the health of individuals or populations. Wildlife health is determined by the cumulative effects of biological, environmental, and socioeconomic pressures acting on individuals and populations. As such, “health”, when quantified, may be used to indicate resilience that reflects the capacity of wildlife to cope with and respond to natural and anthropogenic challenges. Improved knowledge of the health status of species considered vulnerable or at-risk may, therefore, provides valuable information for wildlife management, conservation assessments, and decision making. For example, Whitehead et al. [4] identified gene expression patterns in killifish (*Fundulus grandis*) that were predictive of exposure to hydrocarbon-like chemicals released in the Deepwater Horizon oil spill. Mancia et al. [5] identified gene expression signatures associated with capture stress in wild dolphins (*Tursiops truncatus*). Miller et al. [6] identified a mortality-specific gene expression signature in ocean-tagged sockeye salmon (*Oncorhynchus nerka*), enabling predictions of spawning success. Tinker et al. [7] included gene expression in an analysis of sea otter population collapse, offering substantive data supporting a controversial predation hypothesis. Broad-scale identification of gene expression patterns can provide mechanistic proxies of health [52]. Identifying causal links between exposure to stressors; gene transcript patterns; and individual, population, and ecosystem health is possible.

## Figures and Tables

**Table 1:** Genes and corresponding functions selected for gentoo- and rockhopper-specific quantitative PCR panel.

Gene	Gene Function	General Category
YWHAZ	Reference gene [19]	Reference
IFIT5	Interferon Induced Protein With Tetratricopeptide Repeats 5 (IFIT5) is part of a novel class of IFN-effectors, known as IFN-induced proteins with tetratricopeptides repeats (IFITs). IFIT proteins are indicative of early response to virus [20,21].	Inflammation
IL-6	Interleukin 6 (IL-6) is a cytokine that stimulates the synthesis of the full spectrum of acute phase proteins as seen in inflammatory states [22]. The term “acute phase response” (APR) is referred to a nonspecific and complex reaction of an organism that occurs shortly after any tissue damage, such as infection, trauma, neoplasia, inflammation, and stress [23].	Inflammation
MHC	Major histocompatibility complex class II beta (MHC) molecules play a key role in the adaptive immune responses of vertebrates. MHC class II beta has primarily been associated with extracellular infections (e.g., bacteria) [24].	Targeted immunity
Nr3c1	Nuclear Receptor Subfamily 3 Group C Member 1 (Nr3c1) is a glucocorticoid receptor expressed in response to stress [25].	Stress response
TNFRSF6	Tumor necrosis factor receptor super family 6 (TNFRSF6) is instrumental in a number of cellular signaling pathways involving inflammation, apoptosis, lymphocyte homeostasis, and tissue development [26]. TNFRSF6 also plays a prominent role in apoptotic clearance of virus-infected cells [27].	Inflammation
AHR	The aryl hydrocarbon receptor (AHR) responds to classes of environmental toxicants including polycyclic aromatic hydrocarbons, polyhalogenated hydrocarbons, dibenzofurans, and dioxin [28]. Birds have been found to have different sensitivities to PHAHs and TCDD exposure in comparison to other species; this can be due to expression differences in AHR [29].	Detoxification
THRa	Thyroid hormone receptor alpha (THRa) is associated with physiological stress and organic compound exposure [30].	Stress response
HSP70	The heat-shock protein 70 (HSP70) is produced in response to exposure to different kinds of environmental stress conditions, such as infection, inflammation, exercise, exposure of the cell to toxins, starvation, and thermal or other stress [31,32]. In addition to being expressed in response to a wide array of stressors, heat-shock proteins act as molecular chaperones [33]. In incubating female eiders, an increase in HSP70 resulted in a decrease of immunoglobulin [34].	Stress response
IL-18	Interleukin-18 (IL-18) plays an important role in inflammation and host defense against microbes. Induction of IL-18 initiates a TH1 immune response in chickens [35,36].	Inflammation
Gata3	Gata3 is a TH2-specific transcription factor that controls transcription of cytokines Interleukin (IL) IL-4, -5, and -13 [37]. Gata3 is involved in innate and adaptive immune responses to parasitic helminths [38]. Gata3 has also been shown to be involved in adipocyte development in Adelie penguin chicks [30].	Innate and adaptive immune function
PRDX4	Peroxiredoxin 4 (PRDX4) protects against oxidative damage by scavenging reactive oxygen species in both the intracellular (especially the endoplasmic reticulum) compartments and the extracellular space [39,40,41].	Oxidative stress response
PRDX6	Peroxiredoxin 6 (PRDX6) plays a role in redox regulation, phospholipid turnover, and protection against oxidative injury [39,40,41].	Oxidative stress response
GHR	Growth hormone receptor (GHR) is associated with nutrition, growth, and is a regulator of aging and plays a significant role in cancer development [42,43]. GHR expression is decreased in association starvation in some species [44].	Nutrition
VEGFA	Vascular endothelial growth factor A (VEGFA) is a cytokine involved in immune suppression [45].	Immune suppression

**Table 2:** Gentoo and Rockhopper penguin-specific quantitative real-time polymerase chain reaction primers.

Gene	Primer Name	FP1	Primer Name	RP1 rc	Expected Amplicon (bp)
AHR	Sphen. AHR F1	aggacgattaaagtttctccat	Sphen. AHR R1rc	gatagatggtggctgcagg	111
IL-18	Sphen. IL18 F1	tgttgtgagaaagaatgtggaa	Sphen. IL-18 R2rc	acttaaatgctctggagctac	133
GATA3	Sphen. GATA3 F1	ggtccatgacaaccttgaag	Sphen. GATA3 R2rc	tgcatcggtgtcggtgtag	137
PRDX6	Sphen. PRDX6 F1	aggacatcaatgcatacaacg	Sphen. PRDX6 R1rc	ccatccttgtcccgctcat	126
GHR	Sphen. GHR F1	gatccaccaccaacagcag	Sphen. GHR R1rc	tggaactattgttgagagcct	122
VEGFA	Sphen. VEGFA F1	gccttgctcagagaggaga	Sphen. VEGFA R1rc	cacatctgcaagtgcgctc	127
Nr3c1	Sphen. Nr3c1 F1	tgcatcgctctctcagcag	Sphen. Nr3c1 R1rc	aaggagctaacgtctcatcc	118
IFIT5	Sphen. IFIT5 F2	ttgccaggagaagtcttgtta	Sphen. IFIT5 R2rc	cttgaaagctttttgcagctg	120
THRa	Sphen. THRa F1	ggcagccactggaagcag	Sphen. THRa R1rc	ctcgctgaacgcctccag	107
PRDX4	Sphen. PRDX4 F1	agcatggattaatactcctcg	Sphen. PRDX4 R1rc	cttggtcttccagatatacac	115
YWHAZ	Sphen. YWHAZ F1	aaggagatgcagccaacaca	Sphen. YWHAZ R1rc	agttcagcaattgcttcatcaa	136
MHC	Sphen. MHC class II F	aacggcaccgagcgggtgaggt	Sphen. MHC class II R	cccgtagttgtgttggcag	198
IL-6	Sphen. IL-6 F1	cacctcatcctccgagact	Sphen. IL-6 R1rc	tgtaacaaaggattgtgcctg	121
TNFRSF6	Sphen. TNFRSF6 F1	aatgtcgggagagactggaa	Sphen. TNFRSF6 R1rc	gaagtgactgagccaactgt	117
HSP70	Sphen. HSP70 F1	gagcacaagcagaaagagct	Sphen. HSP70 R1rc	ttaatctacttcttcgatggtc	119

**Table 3:** Means and (standard deviations) for all variables across all sites and species. Note: higher numbers indicate less expression.

	Gentoo		Rockhopper	
	Falkland-Malvinas Islands *n* = 39	Detroit Zoo *n* = 23	Falkland-Malvinas Islands *n* = 34	Detroit Zoo *n* = 15
**AHR**	3.23 (1.15)	5.90 (1.54)	3.58 (1.08)	8.02 (2.04)
**GATA3**	−0.39 (0.98)	−1.73 (0.44)	−0.30 (0.79)	−0.96 (0.52)
**GHR**	2.16 (1.22)	2.70 (1.46)	6.80 (1.93)	9.60 (3.14)
**HSP70**	6.07 (2.19)	2.40 (1.04)	7.49 (0.83)	4.43 (1.58)
**IFIT5**	1.50 (0.85)	1.86 (0.83)	−2.45 (0.82)	−0.79 (0.74)
**IL-18**	8.31 (1.10)	6.52 (0.96)	9.21 (0.93)	9.71 (1.10)
**IL-6**	4.40 (0.84)	5.78 (0.58)	4.44 (1.27)	6.02 (0.74)
**MHC**	1.53 (1.22)	0.89 (1.25)	2.64 (1.52)	3.13 (1.16)
**Nr3c1**	−2.73 (0.50)	−1.83 (0.64)	−2.57 (0.59)	−0.84 (0.68)
**PRDX4**	−0.54 (0.88)	−0.91 (0.69)	−0.18 (0.72)	0.52 (0.74)
**PRDX6**	−2.00 (0.74)	−2.82 (0.81)	−1.58 (0.62)	−1.91 (0.91)
**THRa**	2.03 (0.93)	0.87 (0.74)	1.82 (0.76)	1.67 (0.71)
**TNFRSF6**	−2.91 (0.50)	−0.62 (0.48)	−2.92 (0.35)	−0.81 (0.46)
**VEGFA**	5.00 (1.76)	4.90 (0.67)	5.52 (1.54)	6.54 (1.00)

**Table 4:** Mixed effects statistical models (corrected for multiple tests) were used to simultaneously estimate and account for multiple influences on gene expression levels. We used linear mixed-effects models to analyze the influence of sex, location, and species on each gene expression level. Parameter estimates for all model effects were calculated using the lme4 package in R 2.8.1 [50]. Significance was determined at *p* ≤ 0.05. *p* values are reported. Location and species had significant effects on the expression levels of most genes, while sex significantly influenced expression levels of 50% of the genes examined.

Gene	Sex	Location	Species
**AHR**	1.45 × 10^−5^	<2.2 × 10^−16^	5.9 × 10^−3^
**GATA3**		5.98 × 10^−10^	1.0 × 10^−1^
**GHR**	8.6 × 10^−3^	1.59 × 10^−5^	<2.2 × 10^−16^
**HSP70**		4.01 × 10^−16^	6.47 × 10^−7^
**IFIT5**	7.2 × 10^−3^	4.34 × 10^−6^	<2.2 × 10^−16^
**IL-18**		8.9 × 10^−4^	6.97 × 10^−12^
**IL-6**	7.7 × 10^−3^	3.11 × 10^−11^	
**MHC**			2.875 × 10^−8^
**Nr3c1**	1.4 × 10^−3^	<2.2 × 10^−16^	2.3 × 10^−3^
**PRDX4**			1.17 × 10^−5^
**PRDX6**		6.0 × 10^−5^	4.16 × 10^−5^
**THRa**		1.49 × 10^−5^	
**TNFRSF6**	3.7 × 10^−4^	<2.2 × 10^−16^	2.1 × 10^−2^
**VEGFA**	4.0 × 10^−2^		1.6 × 10^−3^

## Data Availability

The data presented in this study are available in Supplementary Material Table S1.

## Supplementary Materials

The following supporting information can be downloaded at: https://www.mdpi.com/article/10.3390/life12020258/s1, Table S1: Normalized values of target genes for all samples included in this study.
