# Supplementary material for: Baseline Gene Expression Levels in Falkland-Malvinas Island Penguins: Towards a New Monitoring Paradigm
Source: Life (Basel). 2022 Feb 9;12(2):258. doi: 10.3390/life12020258 (PMC8880734; doi:10.3390/life12020258)
Supplement: Supplementary file 1 [file life-12-00258-s001.zip › life-1469844-supplementary.pdf]

life

MDPI

Supplementary Material of Baseline Gene Expression Levels in Falkland-Malvinas Island Penguins: Towards a New Monitoring Paradigm

Table S1. Normalized values of target genes for all samples included in this study.

Sample ID	Location	Species	Sex	AHR	GATA3	GHR	HSP70	IFIT5	IL_18	IL_6	MHC	Nr3c1	PRDX4	PRDX6	THRa	TNFRSF6	VEGFA
1	Captive	Gentoo	M	5.98	-1.37	0.43	2.11	1.74	4.84	6.16	0.78	-1.65	-1.49	-2.71	0.87	-0.77	5.33
2	Captive	Gentoo	M	6.30	-1.39	0.87	1.62	2.57	6.13	6.91	0.43	-1.27	-1.29	-2.62	1.15	-0.33	5.29
3	Captive	Gentoo	F	5.75	-0.90	3.02	1.37	2.69	6.73	6.06	0.94	-1.17	-0.78	-2.44	1.27	-0.51	4.99
4	Captive	Gentoo	M	5.75	-1.52	2.23	1.75	1.93	5.44	5.24	1.36	-1.97	-0.79	-2.51	0.74	-1.03	4.41
5	Captive	Gentoo	M	6.18	-1.37	2.25	2.16	2.74	7.14	5.79	1.17	-1.62	0.06	-3.01	1.15	0.08	5.07
6	Captive	Gentoo	M	4.83	-1.92	0.57	1.91	0.84	5.23	5.00	-1.20	-2.80	-1.29	-4.34	-0.21	-1.26	3.82
7	Captive	Gentoo	M	5.27	-1.81	1.37	2.96	1.92	6.02	5.63	1.31	-1.99	-0.71	-2.63	1.28	-0.55	4.93
8	Captive	Gentoo	M	6.82	-1.53	2.91	3.04	2.95	6.88	6.58	2.53	-0.83	-1.16	-1.93	2.10	0.21	6.19
9	Captive	Gentoo	M	6.38	-1.82	3.36	3.92	1.15	6.45	6.12	2.94	-1.64	-1.22	-1.58	0.99	-0.67	5.19
10	Captive	Gentoo	M	12.21	-2.35	1.23	1.60	2.09	5.69	4.77	-0.59	-2.61	-1.97	-4.32	-0.44	-1.36	3.63
11	Captive	Gentoo	M	6.30	-1.80	1.65	2.37	0.81	6.89	6.21	1.42	-2.11	-1.43	-3.47	0.34	-1.13	4.11
12	Captive	Gentoo	M	5.21	-1.29	1.05	2.28	1.35	6.10	5.89	2.49	-1.86	-0.62	-2.09	0.30	-0.57	4.76
13	Captive	Gentoo	F	6.58	-1.78	5.11	2.95	1.79	7.56	6.78	2.81	-0.75	-0.85	-1.66	2.23	-0.18	5.88
14	Captive	Gentoo	F	4.44	-2.94	2.14	2.81	-0.81	5.27	5.50	-0.84	-2.73	-2.26	-2.88	-0.13	-0.63	4.42
15	Captive	Gentoo	F	5.31	-1.29	1.67	6.22	1.76	6.71	6.21	1.14	-1.24	0.46	-2.14	1.59	-0.48	5.86
16	Captive	Gentoo	F	6.45	-1.62	4.16	2.15	2.24	7.76	6.33	2.07	-0.68	-0.85	-1.56	2.01	0.09	5.51
17	Captive	Gentoo	F	5.60	-1.53	4.99	2.75	2.41	7.41	5.46	0.68	-1.84	-0.56	-2.75	0.93	-0.54	5.18
18	Captive	Gentoo	F	4.62	-2.33	5.03	1.43	1.31	5.77	5.55	-1.33	-2.10	-1.46	-3.77	1.09	-0.38	4.53
19	Captive	Gentoo	F	5.78	-1.44	3.78	2.44	2.51	7.23	5.74	1.87	-1.77	-0.84	-2.75	1.23	0.04	4.67
20	Captive	Gentoo	F	5.39	-2.00	3.80	2.37	2.11	6.66	5.42	-0.13	-2.34	-1.17	-3.69	0.29	-1.24	5.27
21	Captive	Gentoo	F	4.65	-2.13	4.08	1.88	2.68	6.46	5.01	0.17	-2.32	0.24	-3.17	0.76	-1.21	4.66
22	Captive	Gentoo	F	4.63	-1.98	2.78	1.79	2.19	6.65	5.13	-0.49	-2.94	-1.29	-3.70	-0.28	-1.17	3.90
23	Captive	Gentoo	F	5.35	-1.73	3.67	1.43	1.75	9.07	5.48	1.04	-1.86	0.29	-3.20	0.80	-0.78	5.16
24	Captive	Rockhopper	M	8.37	-0.31	8.76	3.63	0.19	12.82	6.82	4.42	-0.69	0.83	-2.72	1.62	-0.37	8.13
25	Captive	Rockhopper	M	7.56	-0.56	9.88	3.99	-1.19	8.61	5.72	3.18	-0.11	0.79	-1.44	1.88	-0.95	7.31
26	Captive	Rockhopper	F	7.62	-0.87	12.35	5.26	-0.57	10.61	4.82	1.98	-1.84	0.74	-2.96	0.41	-1.05	5.21
27	Captive	Rockhopper	F	6.64	-1.90	13.42	4.71	-2.07	9.02	6.04	1.33	-1.94	-1.09	-1.72	0.61	-1.15	5.97
28	Captive	Rockhopper	F	7.02	-1.13	12.49	8.19	-1.40	8.75	5.69	2.90	-1.59	-0.09	-0.97	1.60	-1.84	7.20
29	Captive	Rockhopper	M	12.13	-0.75	6.70	7.27	-0.93	9.08	6.92	4.91	0.03	1.02	-0.92	2.44	-0.55	7.76
30	Captive	Rockhopper	M	12.95	-1.13	7.72	5.29	0.73	9.40	6.02	2.50	-0.33	1.32	-2.03	2.19	-0.31	6.41
31	Captive	Rockhopper	M	6.53	-1.14	7.42	3.37	-0.05	10.44	5.38	2.28	-1.21	1.74	-2.53	1.55	-1.10	6.33
32	Captive	Rockhopper	M	5.91	-1.43	12.83	4.18	-0.47	9.25	5.32	1.93	-1.42	0.80	-2.60	0.77	-1.21	4.80
33	Captive	Rockhopper	M	8.28	0.09	9.44	4.38	-0.96	9.43	7.61	3.30	-0.87	-0.40	-1.99	1.96	-0.93	7.44
34	Captive	Rockhopper	F	6.03	-1.34	2.25	2.16	-1.26	10.88	5.79	2.71	-1.14	0.99	-3.39	1.21	0.08	5.55
35	Captive	Rockhopper	F	8.05	-1.11	9.96	4.02	-0.65	8.80	6.85	5.46	-0.10	-0.36	-0.25	2.95	-0.56	7.50
36	Captive	Rockhopper	F	6.45	-1.48	14.26	3.08	-0.45	9.61	5.36	3.36	-0.99	0.15	-2.59	1.41	-1.02	5.59
37	Captive	Rockhopper	M	7.93	-0.89	7.89	3.81	-1.83	9.13	6.12	2.63	-0.03	0.47	-1.85	2.20	-0.52	6.77
38	Captive	Rockhopper	F	8.90	-0.36	8.59	3.16	-0.94	9.88	5.88	4.02	-0.32	0.95	-0.70	2.20	-0.70	6.18
39	Islands	Gentoo	M	3.67	-1.08	1.41	4.70	1.78	9.80	4.75	1.26	-2.10	-0.47	-2.80	1.59	-2.57	4.51
40	Islands	Gentoo	F	3.61	0.04	4.64	6.71	2.22	7.72	5.40	0.55	-2.64	1.18	-2.67	2.42	-2.39	3.80
41	Islands	Gentoo	M	4.85	-0.46	0.86	8.05	2.73	8.94	4.77	2.01	-1.97	-0.08	-1.54	2.15	-2.31	4.28
42	Islands	Gentoo	F	2.23	-0.99	2.43	7.59	1.45	7.24	7.43	-0.11	-2.84	-0.10	-1.91	1.94	-2.89	7.59
43	Islands	Gentoo	F	3.39	-1.55	3.33	5.31	2.93	8.24	4.05	0.82	-3.52	-0.82	-3.12	0.78	-3.54	3.29
44	Islands	Gentoo	F	3.69	0.83	2.53	7.85	1.78	10.20	4.98	2.08	-2.70	0.17	-0.63	2.28	-3.05	4.97
45	Islands	Gentoo	F	2.33	-0.27	1.31	7.44	1.43	8.41	4.40	2.60	-2.57	-1.42	-1.34	3.01	-2.72	9.60
46	Islands	Gentoo	M	3.24	-2.09	1.62	6.50	2.34	7.62	3.30	1.90	-3.11	-0.61	-2.29	1.82	-3.05	8.03
47	Islands	Gentoo	F	3.31	-1.28	3.56	2.88	2.40	9.56	3.79	0.77	-3.71	0.87	-3.16	0.83	-2.02	3.87
48	Islands	Gentoo	F	3.36	-1.14	2.04	6.35	0.17	9.59	4.16	-0.59	-2.84	-0.56	-2.52	1.56	-3.00	4.58
49	Islands	Gentoo	M	3.77	0.48	1.59	7.99	2.22	9.52	4.95	1.33	-2.32	0.16	-1.71	2.92	-2.42	3.75
50	Islands	Gentoo	F	3.33	2.03	3.79	2.40	-0.12	8.70	4.79	4.39	-2.57	0.18	-0.77	3.25	-2.86	5.66
51	Islands	Gentoo	F	3.40	0.30	2.24	2.20	1.71	8.64	3.68	1.79	-2.61	-0.81	-2.21	2.50	-2.95	4.87
52	Islands	Gentoo	M	3.58	-1.14	1.54	6.90	1.94	8.63	4.23	1.20	-2.99	1.19	-2.26	1.14	-3.21	3.32
53	Islands	Gentoo	F	4.53	-0.97	2.85	5.37	2.77	8.47	3.58	0.36	-2.98	-0.01	-3.04	0.41	-2.73	4.14
54	Islands	Gentoo	F	3.70	-1.38	3.26	5.72	2.63	9.31	4.58	-1.01	-2.90	0.36	-2.86	0.04	-2.80	3.92
55	Islands	Gentoo	F	4.20	-0.96	3.59	3.70	1.74	9.41	4.92	2.55	-2.71	0.83	-2.09	2.29	-2.65	4.22
56	Islands	Gentoo	M	3.46	-0.68	0.36	7.57	2.51	8.32	4.39	1.69	-2.26	-0.23	-1.62	1.44	-3.04	9.21
57	Islands	Gentoo	F	2.41	-0.11	3.14	8.72	0.28	9.25	4.30	2.20	-2.95	0.24	-2.13	0.70	-2.68	4.75
58	Islands	Gentoo	F	3.73	-0.36	2.28	7.77	1.07	10.21	3.90	4.02	-2.78	-1.04	-1.80	2.90	-4.12	4.96
59	Islands	Gentoo	F	1.90	-0.64	2.21	8.39	0.69	7.62	4.22	1.02	-2.80	-2.10	-1.90	2.30	-3.81	3.36
60	Islands	Gentoo	F	3.40	-1.37	2.88	4.62	2.11	6.45	3.68	0.00	-3.37	-1.01	-3.15	0.35	-3.32	4.86
61	Islands	Gentoo	F	2.94	0.02	2.34	7.73	1.28	9.81	3.62	1.34	-2.59	-1.13	-2.31	2.59	-3.14	4.80
62	Islands	Gentoo	F	3.30	1.04	1.33	8.16	1.68	6.48	3.69	1.70	-2.32	-0.29	-1.18	2.29	-3.57	5.41
63	Islands	Gentoo	F	3.61	-1.02	2.10	5.30	1.94	7.26	4.75	2.37	-2.75	-0.75	-1.20	1.53	-2.29	4.05
64	Islands	Gentoo	M	1.57	0.52	-0.55	4.92	1.33	7.57	3.87	2.93	-2.46	-1.55	-1.67	3.04	-3.46	4.46
65	Islands	Gentoo	F	2.64	0.65	3.01	7.81	1.50	7.99	3.97	1.80	-2.71	-0.48	-1.21	1.86	-2.86	5.32
66	Islands	Gentoo	F	2.67	1.35	3.11	6.30	0.84	7.94	4.84	2.46	-1.88	-0.72	-0.88	2.63	-1.77	4.36
67	Islands	Gentoo	M	2.55	-2.18	-0.06	6.22	1.15	6.60	4.13	1.22	-3.56	-2.17	-2.65	1.85	-3.21	3.29
68	Islands	Gentoo	M	3.45	1.48	1.45	8.57	2.10	9.18	4.58	3.49	-1.48	-0.23	-0.43	4.04	-2.58	6.10
69	Islands	Gentoo	M	3.05	-0.52	-0.49	7.38	1.10	8.25	6.26	2.06	-2.17	-0.79	-1.71	2.38	-3.63	4.41
70	Islands	Gentoo	F	1.95	-0.01	4.70	3.42	1.59	7.97	3.63	1.53	-2.85	-0.91	-2.31	2.35	-2.39	5.02
71	Islands	Gentoo	M	3.48	0.51	1.31	7.94	1.46	9.34	6.15	0.15	-1.87	-0.24	-1.13	2.91	-2.63	6.04
72	Islands	Gentoo	F	2.40	-0.42	3.17	4.40	1.03	7.81	4.75	-0.41	-2.92	-0.28	-2.83	0.38	-2.59	3.79
73	Islands	Gentoo	M	2.82	0.31	1.38	9.16	0.79	7.15	4.32	3.19	-2.71	-1.65	-1.46	3.20	-3.26	10.57
74	Islands	Gentoo	F	8.41	-0.83	2.12	8.41	1.08	8.52	3.34	0.44	-3.66	-0.48	-2.76	1.93	-2.54	4.50
75	Islands	Gentoo	F	1.17	-0.79	2.25	3.44	-1.10	5.76	3.63	1.48	-3.25	-2.48	-2.08	3.05	-3.65	4.55
76	Islands	Gentoo	M	2.24	-1.31	2.28	1.95	0.85	7.35	3.69	2.20	-2.88	-0.92	-1.91	2.33	-2.81	3.23
77	Islands	Gentoo	F	2.52	-1.14	1.46	0.87	1.00	7.36	4.18	0.75	-3.27	-1.97	-2.41	2.24	-3.09	3.42
78	Islands	Rockhopper	F	3.68	0.23	5.20	4.08	-2.21	10.70	3.65	2.97	-3.55	0.36	-2.56	0.80	-2.79	4.24
79	Islands	Rockhopper	F	3.64	0.85	9.74	7.96	-3.16	8.26	3.78	3.74	-2.09	-0.16	-2.04	2.04	-3.10	4.96
80	Islands	Rockhopper	F	2.74	-0.56	7.88	5.90	-2.62	8.51	3.54	0.92	-2.24	1.63	-1.91	1.05	-2.39	5.25
81	Islands	Rockhopper	F	3.92	-0.02	5.95	4.19	-0.77	10.20	3.57	3.32	-2.46	0.83	-2.20	1.54	-2.26	5.72
82	Islands	Rockhopper	M	4.47	-0.24	6.33	8.41	-0.84	8.54	4.32	1.66	-2.77	0.93	-2.84	0.39	-2.75	4.48
83	Islands	Rockhopper	F	4.18	-0.78	5.23	7.54	-2.29	8.69	3.94	1.06	-2.39	-0.48	-1.47	1.35	-2.85	5.27
84	Islands	Rockhopper	F	3.88	-1.80	8.37	6.34	-3.17	8.81	3.86	1.52	-3.02	-0.51	-2.22	0.81	-2.69	5.51
85	Islands	Rockhopper	F	4.20	0.79	5.43	7.81	-2.64	9.08	3.95	2.53	-1.78	-0.31	-1.18	3.04	-2.55	5.24
86	Islands	Rockhopper	F	3.46	-1.25	4.66	3.12	-3.52	9.68	10.00	1.49	-3.53	-0.84	-3.15	0.93	-2.97	4.97
87	Islands	Rockhopper	F	4.05	-1.62	5.84	4.29	-1.94	8.54	4.13	2.24	-2.31	0.00	-2.08	0.39	-2.45	6.37
88	Islands	Rockhopper	M	5.75	0.98	6.33	9.06	-1.49	11.80	4.85	3.37	-2.44	0.63	-0.70	1.55	-2.56	5.11
89	Islands	Rockhopper	F	3.21	0.46	4.94	9.31	-3.88	8.51	4.56	2.84	-1.92	-0.74	-0.72	2.31	-2.88	4.32
90	Islands	Rockhopper	F	2.94	-0.23	6.75	8.38	-3.00	9.27	4.73	3.76	-2.56	-0.37	-1.00	1.78	-2.48	5.05
91	Islands	Rockhopper	F	2.61	-0.66	5.01	7.58	-2.66	8.96	4.76	1.45	-2.49	-1.45	-1.37	2.50	-3.32	6.10
92	Islands	Rockhopper	F	3.07	-0.33	9.05	9.39	-2.93	10.11	3.70	1.49	-2.94	0.06	-1.42	1.57	-3.32	4.51
93	Islands	Rockhopper	F	3.79	-1.41	6.02	3.02	-2.19	9.47	4.74	2.23	-2.75	0.27	-1.40	2.10	-2.58	4.82
94	Islands	Rockhopper	F	2.96	-0.01	9.53	8.47	-1.86	7.23	4.09	2.69	-2.18	-0.59	-1.04	3.15	-3.19	5.98
95	Islands	Rockhopper	F	3.56	-0.62	5.01	7.50	-1.84	9.40	3.98	3.86	-2.31	0.37	-0.83	2.48	-2.62	5.68
96	Islands	Rockhopper	M	2.99	0.65	4.89	9.63	-1.64	9.64	4.50	1.36	-2.35	-0.64	-1.61	1.52	-3.36	5.09
97	Islands	Rockhopper	M	3.30	0.43	10.28	8.16	-1.81	10.65	4.44	3.15	-1.86	0.65	-1.30	1.80	-2.98	10.27

98	Islands	Rockhopper	F	3.56	-0.52	4.89	4.62	-2.79	10.28	4.83	2.08	-1.00	-1.24	-1.73	2.10	-2.67	4.79
99	Islands	Rockhopper	M	4.21	1.07	8.67	9.24	-2.51	9.24	4.41	9.24	-2.53	0.37	-0.85	2.33	-2.91	6.17
100	Islands	Rockhopper	F	3.45	-0.09	4.66	5.12	-2.02	10.25	3.82	1.40	-2.69	-0.70	-1.45	1.90	-3.04	4.58
101	Islands	Rockhopper	F	2.50	0.05	8.58	10.15	-2.80	9.24	4.35	2.01	-2.75	-0.32	-1.29	2.19	-3.22	5.47
102	Islands	Rockhopper	F	3.36	0.87	10.58	7.59	-1.35	8.83	4.57	2.41	-1.67	0.41	-0.94	3.28	-2.44	5.34
103	Islands	Rockhopper	M	2.92	-0.27	9.08	9.24	-2.19	9.24	3.77	5.02	-3.21	-0.81	-2.29	1.92	-2.71	9.24
104	Islands	Rockhopper	F	4.27	-0.96	9.41	9.41	-2.57	9.82	3.47	2.38	-2.96	-0.64	-1.48	1.26	-3.33	3.78
105	Islands	Rockhopper	F	2.45	-0.57	4.73	8.31	-3.41	7.98	4.55	2.14	-3.28	-0.86	-1.94	2.22	-3.00	4.13
106	Islands	Rockhopper	F	3.19	-0.74	4.67	7.86	-1.47	9.39	4.35	3.30	-2.23	0.62	-1.56	1.24	-3.03	5.25
107	Islands	Rockhopper	M	2.61	-0.30	5.63	10.05	-3.24	8.82	4.23	1.36	-2.11	-0.97	-1.50	1.91	-3.27	4.03
108	Islands	Rockhopper	M	2.68	-0.77	7.97	7.81	-4.57	8.01	3.43	2.48	-3.51	0.04	-2.01	1.63	-3.75	7.81
109	Islands	Rockhopper	M	8.23	-0.81	4.76	8.23	-2.45	8.46	8.23	2.09	-3.43	-1.49	-1.35	2.93	-3.00	4.13
110	Islands	Rockhopper	F	2.78	-1.96	8.14	8.05	-2.89	9.52	4.12	4.21	-3.34	-0.30	-1.99	1.01	-3.38	9.60
111	Islands	Rockhopper	M	3.11	-0.09	7.06	8.77	-2.76	7.92	3.86	2.05	-2.76	0.26	-0.52	2.84	-3.28	4.59
